# The Biphasic Effect of Lipopolysaccharide on Membrane Potential

**DOI:** 10.3390/membranes15030074

**Published:** 2025-03-02

**Authors:** Maria E. Hadjisavva, Robin L. Cooper

**Affiliations:** Department of Biology, University of Kentucky, Lexington, KY 40506-0225, USA; maria.hadjisavva@uky.edu

**Keywords:** bacteria, endotoxin, K2P, lipopolysaccharide, membrane potential, NALCN, potassium channel, septicemia, sodium channel

## Abstract

Lipopolysaccharide (LPS) from certain strains of Gram-negative bacteria can induce a rapid (<1 s) hyperpolarization of membrane potential, followed by a gradual depolarization exceeding the initial resting membrane potential. Through overexpression of a *Drosophila* ORK1 two-pore-domain K^+^ channel (K2P) in larval muscles and altering the external concentrations of K^+^ and Na^+^ ions, it is clear that the hyperpolarization is due to activating K2P channels and the depolarization is due to promoting an inward Na^+^ leak. When the external Na^+^ concentration is negligible, the LPS-delayed depolarization is dampened. The hyperpolarization induced by LPS can exceed −100 mV when external K^+^ and Na^+^ concentrations are lowered. These results indicate direct action by LPS on ion channels independently of immune responses. Such direct actions may need to be considered when developing clinical treatments for certain forms of bacterial septicemia.

## 1. Introduction

Approximately 1.7 million adults in the United States develop sepsis annually. Although data are not available for worldwide analysis, it is likely to be a significant worldwide issue. In 2022, a total of 350,000 of the adults who developed sepsis in the United States died during their hospitalization [1]. Common culprits of Gram-negative bacterial septicemia are *Escherichia coli*, *Pseudomonas aeruginosa*, *Serratia marcescens*, *Klebsiella pneumoniae* and *Proteus mirabilis* [2]. Gram-negative bacteria can trigger an immune response from the host by secreting endotoxins (i.e., lipopolysaccharides, LPS, and repeats-in-toxin, RTX) [3,4]. LPS is known as a primary cause of the induced immune response in mammals resulting in bacterial infection. This leads to a rise in circulating cytokines. High levels of cytokines generate abnormal neural and cardiac function, becoming harmful to the host [5,6,7,8]. Skeletal and cardiac muscle can be severely impacted by the body’s secondary release of proinflammatory cytokines such as from NF-ĸB, TNF-alpha, IL-1, or IL-6 [9,10,11,12,13].

Therapeutic treatments for bacterial sepsis are typically performed through using antibiotics; however, the lysis of bacteria can result in a surge of cytokines following the release of LPS. Instead of focusing on treating responses to cytokines, the receptors for LPS on cells could be targeted to reduce the production of cytokines. However, this has not been effective since there are no known therapeutic selective blockers for the LPS receptors. The LPS receptor is also complex, as it is known to bind to a Toll-like receptor 4 (TLR4) and associated components, referred to as CD14/TLR4/MD2 complex [11,14,15]. Gene therapy to reduce expression of the CD14/TLR4/MD2 receptor complex has not been sanctioned for humans to reduce the inflammatory response. Thus, the search continues for rapid and reversible pharmacological approaches to block the direct action of LPS; however, it is still not fully understood what the direct actions of LPS are on cellular membranes via ion channels, ion pumps, ionic exchangers, and gene regulation separate from the cellular responses to produce cytokines. Thus, to fully understand the effects of LPS, direct actions on targets other than the CD14/TLR4/MD2 receptor complex need to be addressed, such as caspase 11 and ion channels [16].

The *Drosophila* model has served a vital role in addressing the actions of the immune response induced by LPS and understanding the mechanisms of this action in mammals. This work contributed to the awarding of the Nobel Prize in Physiology or Medicine in 2011 to Bruce A. Beutler and Jules A. Hoffmann [17]. *Drosophila* continues to serve as a proof of concept for many physiological studies related to humans [18,19]. Even though the Toll receptors were first characterized in *Drosophila melanogaster*, the immune response in *Drosophila* is not fully mediated by these receptors [20]. As in mammals and insects in general, the acute and direct action of LPS on tissues has not yet been well studied. Within a second of LPS exposure to body wall muscle of *Drosophila*, as well as crustaceans, the membrane potential transiently hyperpolarizes [20]. Interestingly, it appears that for *Drosophila* muscle, LPS also blocks the glutamate receptors at the neuromuscular junction [20], which are pharmacologically classified as a quisqualate subtype [21]. These reported responses are for strains of LPS isolated from *Serratia marcescens* and *Pseudomonas aeruginosa*. Other forms of LPS from various bacterial strains remain to be investigated for their direct actions on membranes.

At the larval *Drosophila* NMJs, the evoked excitatory potentials (EJPs) and single quantal responses gradually decrease in amplitude with 250 µg/mL LPS and rapidly decrease to then disappear at 500 µg/mL LPS exposure. The preparation is also unresponsive to application of glutamate, thus suggesting a direct blockage of the glutamate receptors by LPS [22]. It has recently been shown that the rapid (i.e., within a second) hyperpolarization by LPS of the muscle in larva *Drosophila* is blocked by doxapram [20,23,24]. Doxapram is known to block TASK K2P (i.e., two-pore-domain K^+^ channel) channels in mammals [25,26,27,28]. Additionally, the TASK subtype is pH-sensitive, as is the membrane potential of the larval muscles. Also, it was recently shown that fluoxetine depolarizes the larval muscle, indicating a block of endogenously expressed K2P channels in this tissue [29]. Overexpression of the *Drosophila* ORK1-K2P channel subtype in muscle resulted in the membrane’s potential to become hyperpolarized, demonstrating the role of K2P channels being responsible for the resting membrane potential [20]. K2P channels are diverse in nature and affect various cells differently, but all appear to affect membrane potential [30,31,32,33,34,35,36].

There are estimated to be 11 known K2P subtypes in *Drosophila* based on genome sequencing; however, their expression profiles in the various tissues are unknown [37,38]. The previous investigations into the acute effect of LPS on the larval *Drosophila* muscle mainly focused on the rapid hyperpolarization being potentially related to transiently activating K2P channels. The hyperpolarization drives the membrane potential towards the equilibrium of the K^+^ ion for the muscle. It was reported earlier that the adult *Drosophila* muscle and moth muscle have an equilibrium potential for K^+^ which are more negative than −90 mV [39,40]. Since the effects were only transient for a few seconds prior to the membrane potential starting to depolarize, it was assumed that the K2P channels desensitized to the LPS, as well as possibly that the Na-K ATPase pump aided in resetting the membrane potential [41]. However, if LPS is not flushed off the muscle, the membrane will continue to depolarize past the original resting membrane potential prior to LPS exposure. If the LPS exposure is short (i.e., about a minute) and flushed with fresh saline several times, then the membrane potential will regain the original resting level and repeated exposure to LPS can induce a similar response as the first exposure [42]. Previously, our research group considered that the continued depolarization induced by the LPS was due to the membrane integrity being compromised without considering that LPS could potentially be activating a Na^+^ leak or voltage-gated Na^+^ and/or Ca^2+^ channels. However, previous studies indicated that the larval body wall muscle of *Drosophila* does not have voltage-gated Na^+^ channels [43,44]. In addition, previous studies have shown that LPS is not hyperactivating the Na-K-ATPase pump in the larval *Drosophila* muscle [45]. Thus, the goal of this current study was to address the mechanisms behind the hyperpolarization and depolarization induced by LPS.

## 2. Results

**Figure 1:**
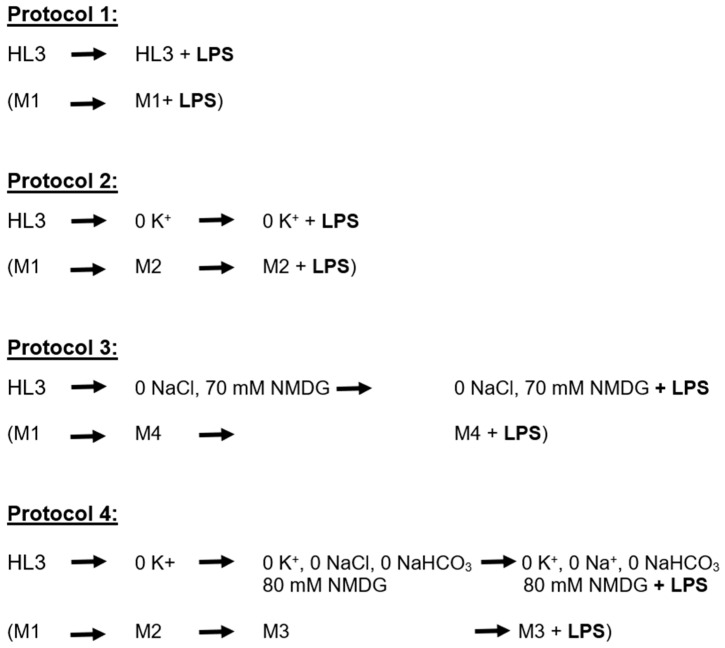
The four protocols used in exchanges of bathing saline to examine the effects of LPS on the membrane potential. The composition of the salines is shown in Table 1. The media used is stated below the described saline (M represents the media type in Table 1).

To determine if LPS was driving the membrane potential towards the equilibrium potential (E_K_) for K^+^ ions, the E_K_ was adjusted by altering the external K^+^ concentration ([K^+^]_o_) as well as the external Na^+^ concentration ([Na^+^]_o_). The different paradigms used for cells with the normal expression of ORK channels and ones overexpressing ORK channels aided in determining whether LPS would have different actions on cells, which would have a membrane potential closer to E_K_ from the overexpression of these leak K^+^ channels and a maximum difference in the concentration difference from external and internal concentrations of K^+^ and Na^+^ ions. In addition, reducing the [Na^+^]_o_ in some of the protocols aided in helping to determine the proposed delayed actions of LPS in depolarizing the membrane though a Na^+^ leak, presumably sodium leak channels (i.e., NALCN channels). The protocols utilized are shown in Figure 1 and the details of each saline solution are listed in Table 1 in the Materials and Methods (Section 4). The description of the results below follows the order of the protocols as normal physiological saline to reduced [K^+^]_o_, and then reduced [Na^+^]_o_ followed by reduced [K^+^]_o_ and [Na^+^]_o_.

**Figure 2:**
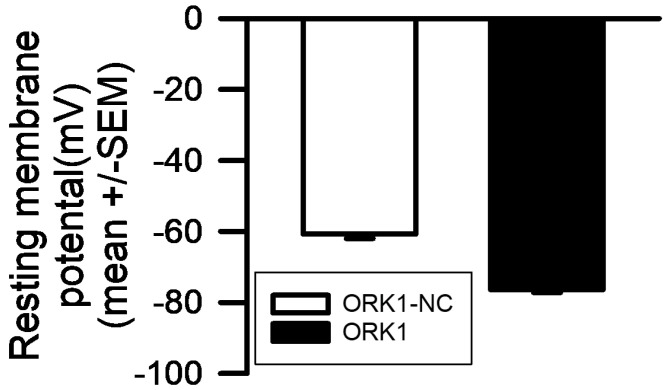
The resting membrane potential differences between ORK1-NC and ORK1 in normal HL3 saline for all muscle fibers examined. M6-M7 > ORK1NC (−60.6 mV +/− 1.4; mean +/− SEM; N = 31) and M6-M7 > ORK1 (−76.17 +/− 1.1; mean +/− SEM; N = 24) for the initial resting membrane potential values measured in this study (*p* = 2.47 × 10^−11^; two-tailed *t*-test).

**Figure 3:**
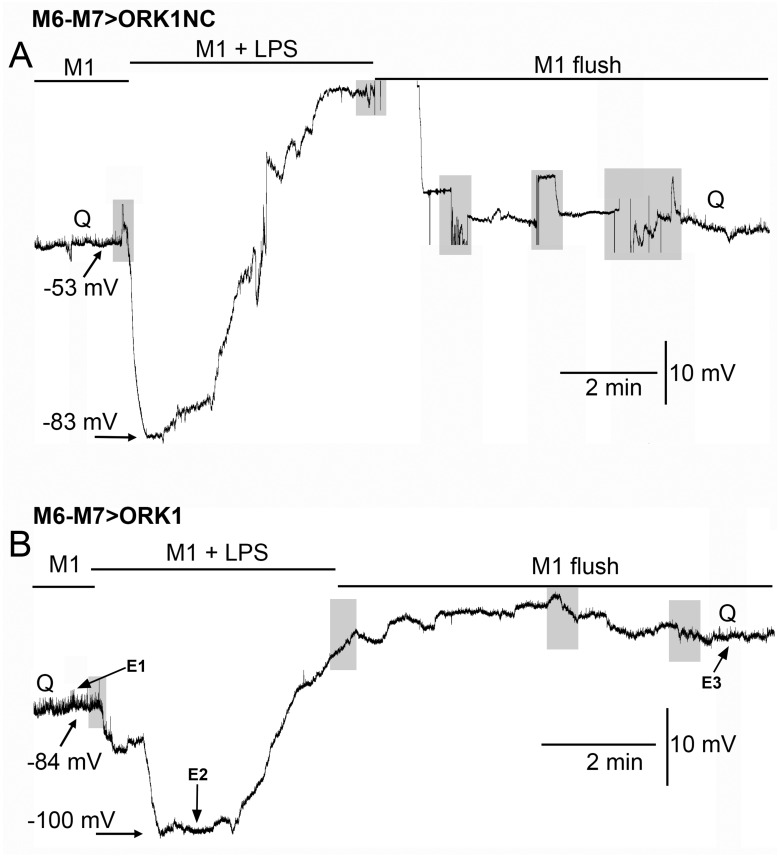

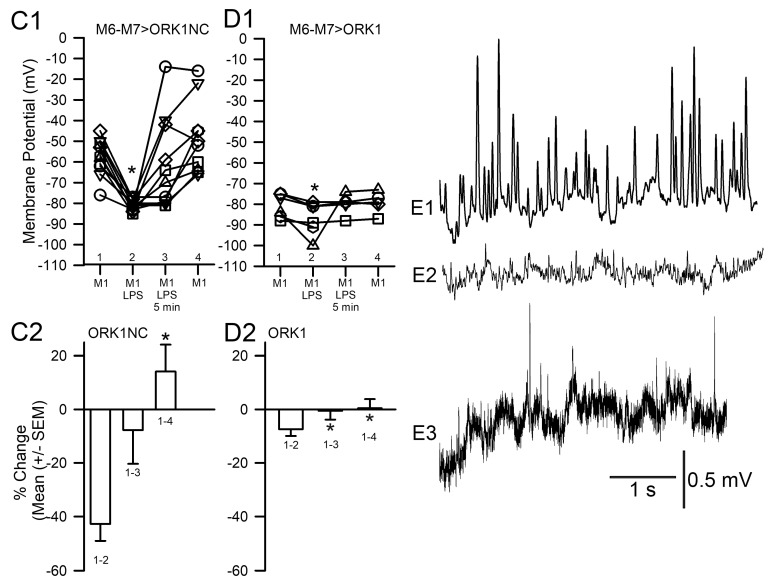
The effect of LPS on membrane potential with the standard HL3 saline (M1, Protocol 1) in control larvae and larvae overexpressing ORK1 channels in muscle. (**A**) A representative recording of the membrane potential from muscle fiber m6 in the M6-M7 > ORK1NC strain before, during, and after exposure to LPS. There was rapid hyperpolarization with LPS, followed by a pronounced depolarization. The quantal events, denoted by Q, are present before exposure to LPS and after multiple flushes to remove the LPS, but are absent during LPS exposure, even with a stronger driving gradient for Na^+^ flux through the ionotropic glutamate receptors. (**B**) The same paradigm as for A but with the M6-M7 > ORK1 strain. Note the initial resting membrane potential was more negative and the extent of change with LPS was not as large due to already starting at a more negative membrane potential. The quantal events again disappear in the presence of LPS. (**C1**,**D1**) The membrane potential values for individual preparation before and during LPS exposure for the most hyperpolarized state obtained over the 5 min of exposure. There was a significant difference in the initial membrane potentials for the two strains (*t*-test, * *p* < 0.05; and within each strain for the effect of LPS (paired *t*-test, * *p* < 0.05). (**C2**,**D2**) The percent change in the membrane potentials from before exposure to LPS for the two illustrated strains. The M6-M7 > ORK1NC strain has a large degree of hyperpolarization as compared to the M6-M7 > ORK1 strain (*t*-test, * *p* < 0.05). The percentage change from the initial values to LPS was significantly different for the initial value and flushing off LPS for ORK1NC and ORK1 ((**C2**,**D2**) ANOVA * *p* < 0.05) and for the percentage change for the initial level to LPS exposure to 5 min exposed to LPS (**D2**) (ANOVA * *p* < 0.05). The shaded boxes illustrate changes in the bathing media. The (**E1**–**E3**) marked on the trace in B was enlarged to illustrate the reduction in the occurrence of minis (i.e., spontaneous quantal events) during LPS exposure and the reappearance after washout of LPS. * represent *p* < 0.05; the line plots in **C1** and **D1** represent individual preparations with various symbols.

**Figure 4:**
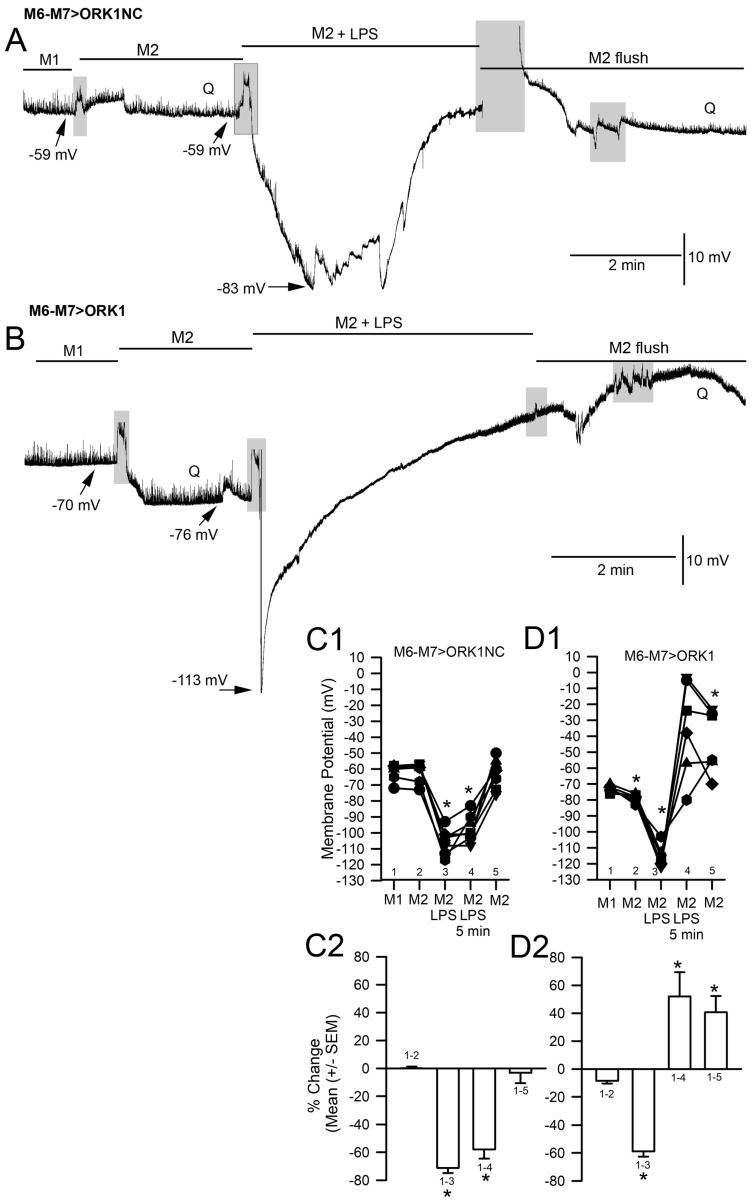
The effect of LPS on membrane potential with the saline without KCl added (M2, Protocol 2) in control larvae and larvae overexpressing ORK1 channels in muscle. (**A**) A representative recording of the membrane potential from muscle fiber m6 in the M6-M7 > ORK1NC strain before, during, and after exposure to LPS. There was rapid hyperpolarization with LPS, followed by a pronounced depolarization. The quantal events, denoted by Q, are present before exposure to LPS and after multiple flushes to remove the LPS, but are absent during LPS exposure. (**B**) The same paradigm as for A, but with the M6-M7 > ORK1 strain. Note the initial resting membrane potential is more negative and the extent of change with LPS is not as large due to already starting at a more negative membrane potential. The quantal events again disappear in the presence of LPS. (**C1**,**D1**) The membrane potential values for individual preparation before and during LPS exposure for the most hyperpolarized state obtained over the 5 min of exposure. There was a significant difference in the initial membrane potentials for the two strains (*t*-test, * *p* < 0.05) and within each strain for the effect of LPS (paired *t*-test, * *p* < 0.05). In addition, the effect of lowered KCl in the saline produced a significant decrease in the membrane potential for the M6-M7 > ORK1 (paired *t*-test, * *p* < 0.05). (**C2**,**D2**) The percent change in the membrane potentials from the initial saline (M1) to exposure of M2 media and from M1 exposure to LPS in the M2 media for the two strains illustrated that the M6-M7 > ORKNC strain has a larger degree of hyperpolarization as compared to the M6-M7 > ORK1 strain for exposure to lowered KCl as well as to LPS (*t*-test, * *p* < 0.05). The shaded boxes illustrate changes in the bathing media. * represent *p* < 0.05; the line plots in **C1** and **D1** represent individual preparations with various symbols.

**Figure 5:**
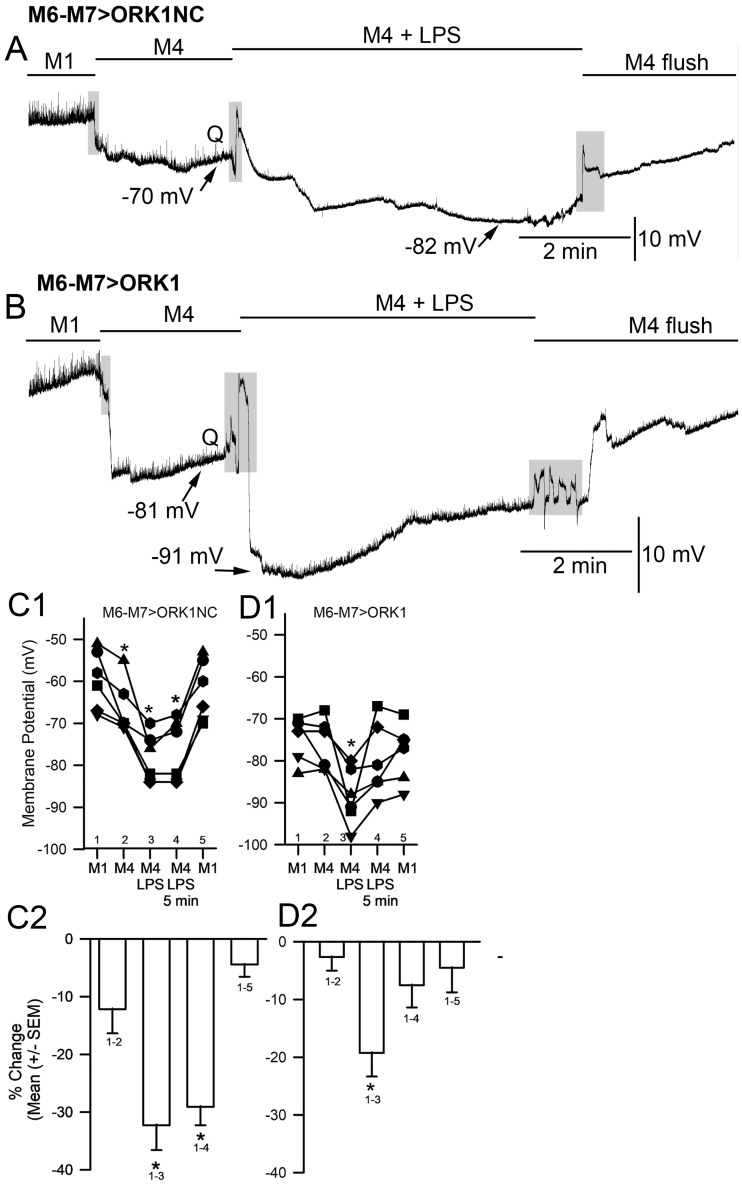
The effect of LPS on membrane potential with the saline where NaCl was substituted for NMDG (M4, Protocol 3) in control larvae and larvae overexpressing ORK1 channels in muscle. (**A**) A representative recording of the membrane potential from muscle fiber m6 in the M6-M7 > ORK1NC strain in saline containing NaCl, and then in one where it was substituted for NMDG followed by exposure to LPS in the low Na^+^ concentration media (M4). There was rapid hyperpolarization in switching from M1 to M4 media, as well as M4 media with LPS. However, there was minimal depolarization over the 5 min of exposure to LPS. The quantal events, denoted by Q, are present before exposure to LPS but are absent during LPS exposure and tend to decrease in amplitude while exposed to M4 media before LPS exposure. They do not appear to return after flushing away LPS, as M4 media were low in Na^+^. (**B**) The same paradigm as for A, but with the M6-M7 > ORK1 strain. Note the initial resting membrane potential was more negative and the extent of change with LPS was not as large due to already starting at a more negative membrane potential. The quantal events again disappear in the presence of low Na^+^ or LPS. (**C1**,**D1**) The membrane potential values for individual preparation before and after exposure to low Na^+^ containing media (M4) and during LPS exposure for the most hyperpolarized state obtained over the 5 min of exposure. There was a significant difference in the initial membrane potentials for the two strains (*t*-test, * *p* < 0.05) and within each strain for the effect of LPS (paired *t*-test, * *p* < 0.05). In addition, the effect of lowered NaCl in the saline produced a significant decrease in the membrane potential for the M6-M7 > ORK1NC (paired *t*-test, *p* < 0.05). (**C2**,**D2**) The percentage change in the membrane potential from the initial saline (M1) to exposure of M4 media and then M1 saline to exposure of LPS in the M4 media for the two strains illustrated that the M6-M7 > ORKNC strain has a larger degree of hyperpolarization as compared to M6-M7 > ORK1 strain for exposure to lowered NaCl as well as to LPS (*t*-test, * *p* < 0.05). (**C2**) represents the percentage change from the initial saline to low Na^+^ and to LPS exposure, as well as after 5 min of exposure to LPS. All of which show a significant difference to the initial change from HL3 saline to low Na^+^ (ANOVA * *p* < 0.05). The shaded boxes illustrate changes in the bathing media. * represent *p* < 0.05; the line plots in **C1** and **D1** represent individual preparations with various symbols.

The first protocol was used to establish the effect of LPS on both M6-M7 > ORK1NC as well as muscles overexpressing the conducting a form of *Drosophila* K2P channels (M6-M7 > ORK1). The use of examining the non-conducting M6-M7 > ORK1NC was to serve as a control in overexpressing proteins and their insertion in the membrane, but not to conduct K^+^ ions. The overall resting membrane potential in the M6-M7 > ORK1 strain was more hyperpolarized. This was also determined by combining data for all four protocols in the membrane potentials of M6-M7 > ORK1NC (−60.6 mV +/− 1.4; mean +/− SEM; N = 31) to compare to M6-M7 > ORK1 (−76.17 +/− 1.1; mean +/− SEM; N = 24) in the initial resting membrane potential values measured within this study (*p* = 2.47 × 10^−11^; two tailed *t*-test; Figure 2, Figure 3, Figure 4 and Figure 5). The resting membrane potential was more negative for the M6-M7 > ORK1, as expected, for constituently active K2P channels [29].

Upon exposure of LPS to the larval muscle of M6-M7 > ORK1NC or M6-M7 > ORK1, for protocol 1, the membrane potential showed a larger hyperpolarized membrane potential when exposed to LPS for both larval strains (Figure 2, paired *t*-test *p* < 0.05) and the degree of hyperpolarization between the two strains to LPS had a slightly more negative potential for M6-M7 > ORK1 (−86.8 mV +/− 8.1, mean +/− SEM; N = 6) compared to M6-M7 > ORK1NC (−81.1 mV +/− 2.7, mean +/− SEM; N = 10) (two-tailed *t*-test, *p* = 0.055).

In previous investigations examining the effect of LPS from *S. marcescens*, the exposures were kept to 1 or 2 min [20,46]; however, for these studies, the exposures were kept at 5 min to examine the extent of depolarization after the initial acute hyperpolarization induced by LPS. For both M6-M7 > ORK1 and M6-M7 > ORK1NC strains, there was a rapid hyperpolarization induced by exposure to LPS, and within a minute, the membrane potential was already depolarized from the hyperpolarization (Figure 3). The amount of change in the membrane potential was greater for the M6-M7 > ORK1NC than M6-M7 > ORK1 with exposure to LPS (Figure 3C1,D1; *t*-test *p* < 0.05). The membrane potential would continue to depolarize to a greater extent than that of the original potential prior to the exposure of LPS. The depolarization was also larger for M6-M7 > ORK1NC, likely due to not having as many K2P channels to maintain the membrane potential closer to E_K_. During the duration of LPS exposure, the depolarization for ORK1NC and ORK1 is substantial, as well as after flushing away the LPS with fresh saline without LPS, as observed in the percentage changes (C2 and D2; ANOVA *p* < 0.05). The E1–E3 marked on the trace in Figure 3B was enlarged to illustrate the reduction in the occurrence of minis (spontaneous quantal events) during LPS exposure and the reappearance after washout of LPS. When LPS was exposed to the muscle fibers, spontaneous quantal events decreased in amplitude and became undetectable, and would sometimes reappear, flushing the LPS off the preparation, as observed in Figure 3E1–E3.

To examine the effect of reducing [K^+^]_o_ on the membrane potential for both M6-M7 > ORK1NC and M6-M7 > ORK1, Protocol 2 was used. There was a significant effect for the M6-M7 > ORK1 (paired *t*-test *p* < 0.05) but not M6-M7 > ORK1NC (Figure 4C1,D1). By lowering the [K^+^]_o_, the E_K_ should be lowered by driving the membrane potential to a more hyperpolarized state and reducing the effect of LPS exposure. Upon exposure to LPS in saline without KCl, the degree of change in hyperpolarization for Protocol 2 was reduced for the M6-M7 > ORK1, since the membrane potential was already hyperpolarized compared to the M6-M7 > ORK1NC strain. The hyperpolarization reached an astonishing level in the range of −110 to −120 mV, with LPS indicating a drive to a more negative E_K_. After the rapid hyperpolarization, the membrane potential would start to depolarize and would continue to do so, even passing the initial resting membrane potential in some cases. This depolarization was assumed to be due to a leak of Na^+^, which was examined in subsequent experimental paradigms. In the saline containing no KCl, the quantal events were detectable, but upon LPS exposure, they would become smaller and be undetectable, although they could reappear by flushing away the LPS, even in saline free of KCl.

To examine the effect of Na^+^ contributing to the repolarization after the initial hyperpolarization, the [Na^+^]_o_ was reduced by not adding NaCl but compensating the reduction in osmolality by adding in the same molarity of NMDG (Protocol 3; Figure 1 and Table 1). In reducing leaks of Na^+^, through potential NALCN channels, the membrane potential was expected to be more hyperpolarized throughout the exposure of LPS.

In replacing the NaCl in the saline, there was a larger hyperpolarization for M6-M7 > ORK1NC than for M6-M7 > ORK1 using Protocol 3 and the M4 saline (Figure 5; paired *t*-test, *p* < 0.05), which seems logical as M6-M7 > ORK1 already has a more negative membrane potential. Upon LPS exposure, both strains again showed a rapid hyperpolarization, but this time, over a 5 min exposure to the membrane potential, did not start to depolarize to the same extent as in the presence of NaCl. The extent of hyperpolarization was greater for the M6-M7 > ORK1NC strain than M6-M7 > ORK1 (Figure 5C1 compared to D1, *t*-test *p* < 0.05). As with NaCl-containing saline (M1) or with KCl-absent (M2 saline), LPS exposure to the spontaneous quantal events decreased in amplitude and became undetectable. However, it can be noted that in the M4 saline with reduced NaCl, the quantal responses decreased in amplitude as expected, due to a decrease in the Na^+^-driving gradient (Figure 3A,B). Upon flushing the LPS off the preparation with this same saline not containing NaCl, the quantal event did not reappear as there was not enough Na^+^ to flux through the glutamate receptors. This was the case for the six preparations of M7 > ORK1NC and the six of M6-M7 > ORK1 (N = 12, Wilcoxon sign rank test; *p* = 8.9 × 10^−10^). Since the glutamate receptors are ionotropic quisqualate receptors, this is expected.

In addition, upon flushing away the LPS with M4 saline, the membrane potential did not depolarize to as large of an extent as for Protocol 2, which contained NaCl.

**Figure 6:**
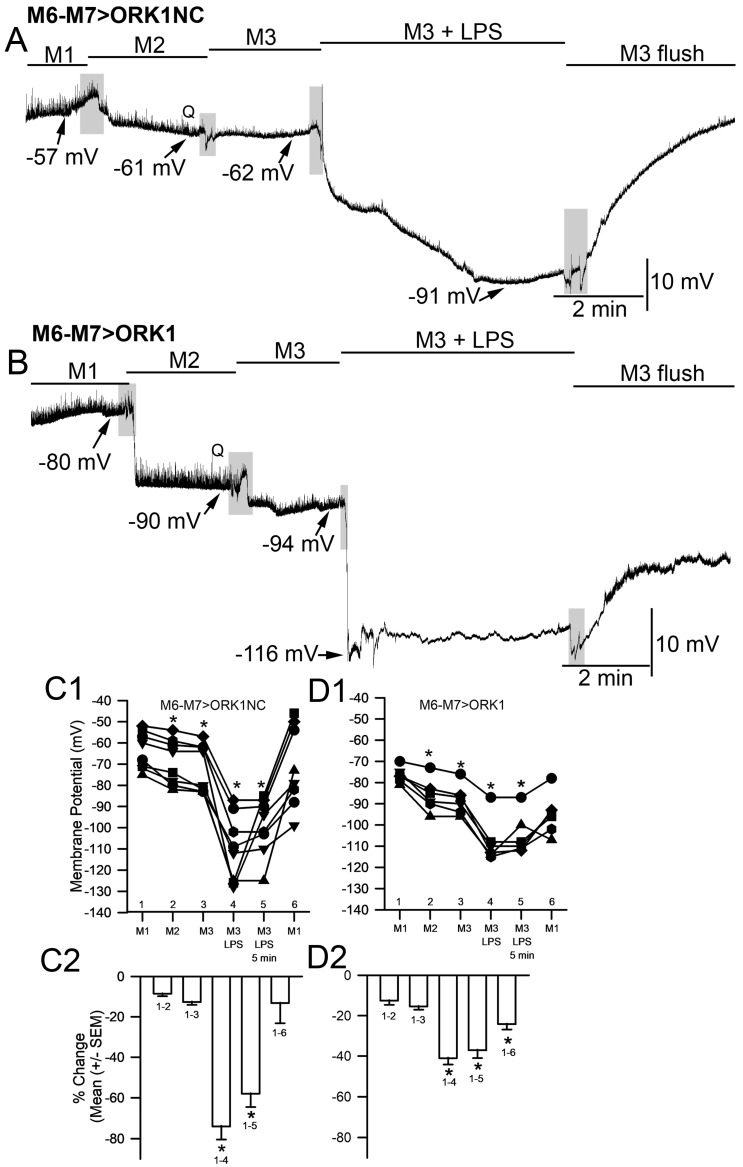
The effect of LPS on membrane potential with the saline where KCl and NaCl were kept to a minimal concentration (M3, Protocol 4) in control larvae and larvae overexpressing ORK1 channels in muscle. (**A**) A representative recording of the membrane potential from muscle fiber m6 in the M6-M7 > ORK1NC strain in saline without KCl and then in saline where no KCl, NaCl, or NaHCO_3_^−^ were added, but NMDG was added to compensate for the low NaCl. This solution was then used to expose the muscles to LPS (M3 + LPS). There was a rapid hyperpolarization in switching from M1 to M2, and then again from M2 to M3 media, as well as from M3 to M3 + LPS media. During exposure to LPS, over the 5 min, there was minimal depolarization. The quantal events, denoted by Q, are present in the exposure to M2 and rapidly disappear when exposed to the M3, and are absent with exposed to LPS and do not reappear after flushing away LPS, as M3 media was very low in Na^+^. (**B**) The same paradigm as for A, but with the M6-M7 > ORK1 strain. Note the initial resting membrane potential was more negative and the extent of change with LPS was not as large due to already starting at a more negative membrane potential. The quantal events again disappear in the presence of low Na^+^ or LPS. (**C1**,**D1**) The membrane potential values for individual preparation before and after exposure to low K^+^- (M2) and then low K^+^- and low Na^+^-containing media (M3) and during LPS exposure for the most hyperpolarized state obtained over the 5 min of exposure. There was a significant difference in the initial membrane potentials for the two strains (*t*-test, * *p* < 0.05; and within each strain for the effect of LPS (paired *t*-test, * *p* < 0.05). In addition, there was a significant effect with lowered KCl, as well as with lowered NaCl together with lowered KCl. There was a significant decrease in the membrane potential for the M6-M7 > ORK1NC and M6-M7 > ORK1 for each saline exchange (paired *t*-test, * *p* < 0.05). (**C2**,**D2**) The percentage change in the membrane potential from M1 to the exposure of M3 + LPS media illustrated that the M6-M7 > ORKNC strain has a larger degree of hyperpolarization as compared to M6-M7 > ORK1 (*t*-test, * *p* < 0.05). The percentage difference from the initial change in M1 to M2 medial to the beginning exposure to LPS and after 5 min M1 to M3 with LPS and to M3 with LPS after 5 min indicates that the membrane remained hyperpolarized during the exposure to LPS. The shaded boxes illustrate changes in the bathing media. The percentage change in the initial values to M1 to M2 (#1 to #2) as compared to the changes in M1 to M3 with LPS (#1 to #4) or M3 with LPS after 5 min (#1 to #5) were significantly different (ANOVA * *p* < 0.05). * represent *p* < 0.05; the line plots in **C1** and **D1** represent individual preparations with various symbols.

In Protocol 3, some Na^+^ remained in the saline because of the presence of NaHCO_3_. To further decrease the extracellular Na^+^ concentration, Protocol 4 was implemented, which excluded both NaHCO_3_ and KCl. This protocol also resulted in prolonged hyperpolarization, with little depolarization for the two *Drosophila* strains with exposure to LPS. The change from media M1 to M2 and M2 to M3 as well as M3 to M3 + LPS all resulted in significantly increased hyperpolarization of the membrane potential (Figure 6A,B,C1,D1; paired *t*-test, *p* < 0.05). As with Protocols 2 and 3, the extent of hyperpolarization for Protocol 4 was greater for M6-M7 > ORK1NC than M6-M7 > ORK1 (Figure 6C2,D2; *t*-test, *p* < 0.05). As expected from the results above, now that [Na^+^]_o_ was reduced as much as possible in the bathing environment, there was little delayed depolarization during LPS exposure and after flushing away the LPS in the M3 media. As expected, the quantal events slowly reduced in amplitude upon switching saline with Na^+^ present to one with low Na^+^; quantal events disappeared altogether during exposure to LPS. As for Protocol 3, the quantal events would not reappear by removing the LPS M1 to LPS and during the low Na^+^ flush. Since the percentage change from the initial change in M1 to M2 media to the beginning exposure to LPS and after 5 min M1 to M3 with LPS and to M3 was large and was not different over the 5 min, this illustrated that in the absence of [Na^+^]_o_, the membrane did not induce a significant repolarization during the LPS exposure.

**Figure 7:**
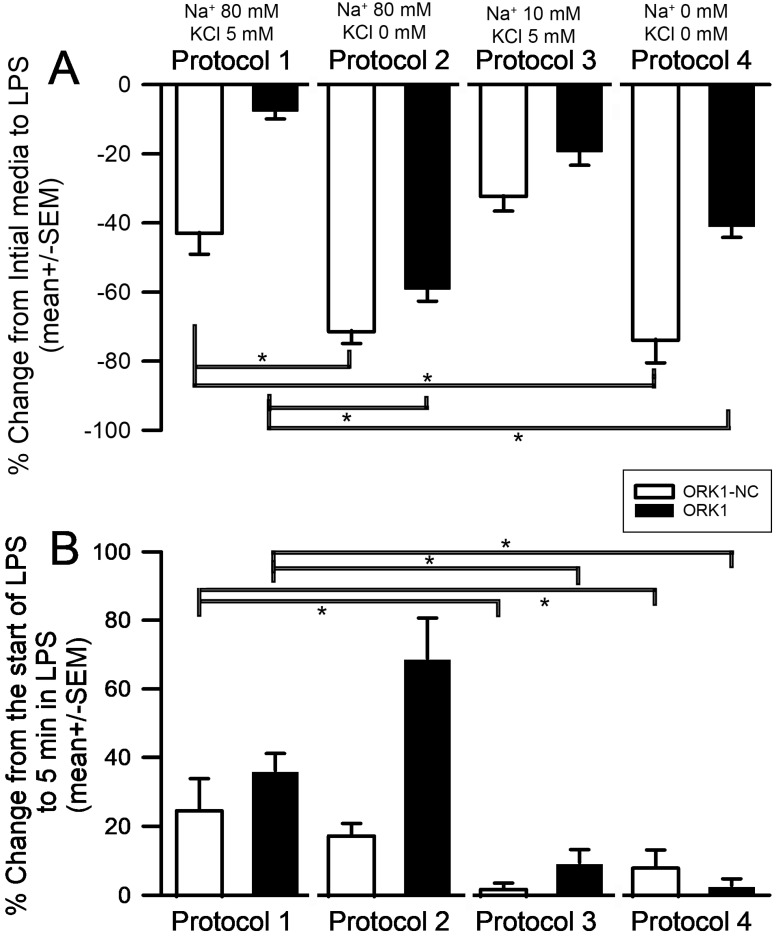
The percentage change in the effect of LPS exposure among the different bathing salines. (**A**) In comparing the differences between ORK1-NC and ORK1, the change of LPS is larger from the initial membrane potential to the hyperpolarized state of LPS for ORK1-NC than ORK1 in all four protocols (*t*-test, *p* < 0.05). Protocols 2 and 4 produced the largest changes due to a lowered [K^+^]_o_ and [Na^+^]_o_ for both ORK1-NC and ORK1 (ANOVA * *p* < 0.05 for each strain). (**B**) In comparing the percentage of change from the start to the end of five minutes in exposure to LPS, Protocols 3 and 4 had the smallest change as compared to Protocol 1 for both ORK1-NC and ORK1 (ANOVA * *p* < 0.05 for each strain).

The differences in the responses to LPS exposure for the different protocols were larger as a percentage change for protocols 2 and 4 as compared to protocol 1 for both ORK1-NC and ORK1 (Figure 7, ANOVA *p* < 0.05 for each strain). This is likely due to the lowered [K^+^]_o_ and [Na^+^]_o_ in the salines. The prolonged exposure to LPS when [Na^+^]_o_ was reduced for protocols 3 and 4 showed the smallest change when compared to protocols 1 and 2 for both ORK1-NC and ORK1 (Figure 7, ANOVA *p* < 0.05 for each strain). The most hyperpolarized value in the membrane potential during LPS exposure was used to determine a percent change to the membrane potential after 5 min of exposure. Percent changes are calculated by: [absolute difference in (value 1–value 2) divided by value 1 and the resultant multiplied by 100]. Negative percent change related to hyperpolarization and positive value related to depolarization from the first measurement.

## 3. Discussion

It is now clear that LPS from certain strains of bacteria initially activate K2P K^+^ channels, driving the membrane towards E_K_. This is followed by an influx of Na^+^, which overrides the K2P activation to result in depolarization. When driving the membrane close to the E_K_ by either overexpressing constituently active K2P channels (i.e., the ORK1) or increasing the driving gradient for K^+^ by lowering the [K^+^]_o_ and then exposing the membrane to LPS, there is not as large an acute hyperpolarization, since the membrane potential is close to E_K_. However, these conditions promote a larger change in depolarization due to Na^+^ influx when Na^+^ is present in the saline. Removing the majority of the external Na^+^ ions from the saline results in retarding the delayed activation of the Na^+^ leak induced by LPS over the 5 min span of exposure. This prolonged hyperpolarization has yet to be noted in prior investigations. Also, the delayed depolarization had not been mechanistically identified as due to Na^+^ influx in prior reports. There are traces of Na^+^ in the saline, even though NaCl was not added due to traces from pH adjustments (NaOH/HCl), the NaHCO_3_ buffer, and likely from trace amounts in the other salts and BES buffer. When the media was used in an attempt to reduce Na^+^ even more without adding NaHCO_3_, the depolarization was even more suppressed, as shown by Protocol 4.

Of the 11 K2P subtypes known to be expressed in *Drosophila*, the extent in the expression profile for the various subtypes is not yet known for any tissue. The overexpression of the ORK1 in the body wall muscle of m6 only added to the endogenous subtypes already expressed and responsible for the maintaining the resting membrane potential. The TASK subtype is blocked by doxapram in mammals and doxapram appears to block not only the endogenous K2P subtypes expressed in *Drosophila* larval muscle, but also the ORK1 subtype when it is overexpressed [23,24,47]. Another pharmacological blocker (i.e., Fluoxetine) of some subtypes of K2P channels, but not necessarily the ORK1 form [29], also depolarizes the larval muscle, indicating that other forms of K2P channel subtypes are expressed in larval muscle. Likewise, chloroform (0.2%) did not block the acute hyperpolarization induced by LPS, and nor did it decrease the delayed depolarization by LPS, but chloroform at 0.2% induced a slight (~2 mV) hyperpolarization and at 2%, induced depolarization with muscle contractions [20]. We are currently conducting studies to address the pharmacological profile of these *Drosophila* ORK1 channels by overexpressing them in the larval muscle and screening actions of various compounds.

It is of interest to better understand the action of LPS on the postsynaptic glutamate receptors in the larval *Drosophila* preparation given that the NMJ of this preparation serves as a model in addressing mechanism of synaptic transmission. Slight Gram-negative bacterial contamination of saline used in physiological studies may well present spurious results due to the actions of LPS. It is known that locomotive behaviors of larvae and adult *Drosophila* are slowed, and survival is reduced when consuming food tainted with LPS [20]. The spontaneous quantal events, as well as evoked excitatory junction potentials, show a depression in amplitude in a dose-dependent manner, with LPS [20,42,46] indicating a gradual blocking of the glutamate receptors, despite an enhanced driving gradient for ions when the membrane is hyperpolarized by LPS. The glutamate receptor subtype at the larval *Drosophila* NMJ is defined as a quisqualate type based on pharmacology and not amino-3-hydroxy-5-methyl-4-isoxazolepropionic acid receptors (AMPA), kainate receptors, or N-methyl D-aspartate (NMDA) subtype, as the receptors are highly sensitive to quisqualate [21]. It would be of interest to investigate if the same strains of LPS also block other glutamate receptor subtypes, as it is suggestive that LPS from *S. marcescens* does have an effect [22].

Even with the delayed depolarization induced by LPS, the spontaneous quantal events, as well as evoked events, are blocked unless the LPS is thoroughly flushed off the preparation [20,42]. The longer that LPS stays on the preparation while Na+ is present and the membrane is depolarized, the less likely it is that flushing off the LPS will restore the normal resting membrane potential. This prolonged exposure appears to permanently compromise the cell membrane, leading to the loss of glutamate receptor function, and ultimately cell death.

This may be due to loading the muscle with Ca^2+^ as the larval muscle uses voltage-gated Ca^2+^ channels on the plasma membrane for inducing muscle contraction. In addition, granulation starts to appear in the muscle fibers, which also occurs with prolonged exposure to cultural media (i.e., Schneider’s and M3) previously used for prolonged physiological studies on larval *Drosophila* preparations [48,49,50,51], which is indicative of the muscle cells dying.

The delayed depolarization from a hyperpolarized state induced by LPS does not appear to be initially due to Ca^2+^ entry, as the depolarization is prevented with reduced Na^+^ alone and the muscle is not contracting. When the membrane is depolarized to a level more depolarized than the normal resting membrane potential, then it is likely the voltage-gated Ca^2+^ channels will come into play. The Na^+^-induced depolarization is gradual, indicating that the response is likely not due to voltage-gated Na^+^ channels but Na^+^ leak channels (i.e., NALCN). In addition, the larval muscle does not appear to express voltage-gated Na^+^ channels [43,44]. NALCN subtypes are diverse in nature and aid in contributing to the membrane potential of cells [52,53,54,55,56,57]. Interestingly, *Drosophila* shares a 57% identity with the human NALCN homolog [56]. Further investigations are warranted into the mechanistic interaction of LPS with K2P and NALCN channels to determine if there is direct interaction with the channels or with accessory proteins for the channels or directly with the bilipid membrane itself, leading to conformational changes in the K2P and NALCN channels. It would be helpful if subtypes of K2P and NALCN channels could be incorporated into reconstituting membranes or artificial membranes to allow their independent functions to be monitored before and after exposure to strains of LPS. Then, various forms of LPS could also be studied to address why different subtypes of LPS produce varied responses. Such interactions of LPS with protein channels are performed in bacterial cells [58]. Potentially optical imaging of labeled LPS would allow one to know if LPS is bound directly to labeled channels and/or accessory proteins [59,60]. Even nanopore technology or measures in membrane dipole potential would be able to assess if channels alter their function directly by interactions of LPS [61,62].

**Figure 8:**
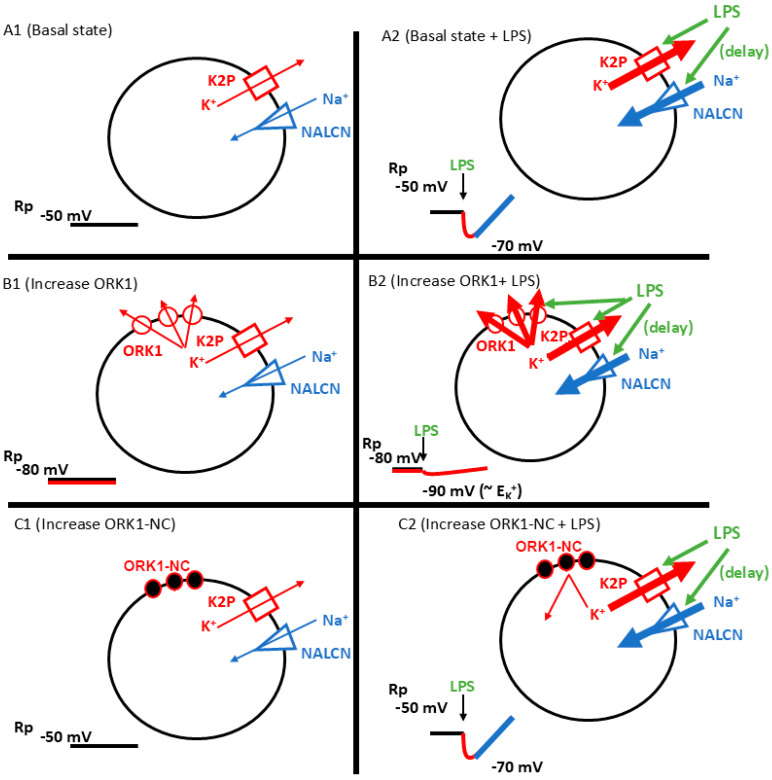
A schematic overview on the effect of overexpression of ORK1 and exposure to LPS on the membrane potential. (**A1**) The basal cell with endogenous natural expression of K2P and NALCN channels to maintain the resting membrane potential (Rp) around −50 mV. (**A2**) The cell’s exposure to LPS initially promotes the opening of the naturally expressed K2P channels, leading to a rapid additional 20 mV hyperpolarization. This is followed by a delayed action of promoting NALCN channels to open, leading to depolarization greater than the initial Rp. (**B1**) A genetically modified cell with overexpression of the ORK1 K2P channel subtype, resulting in a hyperpolarized state (−80 mV) compared to the basal state (−50 mV). (**B2**) A genetically modified cell with an overexpression of the ORK1 K2P channel subtype when exposed to LPS. With the Rp of the cell already at −80 mV, there is a small driving gradient to reach the equilibrium potential for K^+^ (E_K_) when the endogenous natural expression of K2P and the genetically expressed ORK1 channels are enhanced by exposure to LPS. The large flux of K^+^ ions overrides the effect of LPS on the NALCN channels, which leads to a prolonged hyperpolarization close to the E_K_^+^ of the cell. (**C1**) The effect of overexpression of ORK-NC would aid in accounting for effects of overexpressing proteins, and (**C2**) would not promote K^+^ ion flux in the presence of LPS and would be expected to be similar to exposure to LPS for cell in their native state, as shown in A2. (The thickness of the arrows represents the degree of the effect of the ionic flux and the solid fill of the channels represents that they are enhanced by the presence of LPS. The effects on the Rp are schematically illustrated in the bottom left of each cellular condition).

The response times are also of interest to address, as the hyperpolarization is so rapid as compared to the gradual depolarization. Potentially, the binding affinity is higher for the K2P channels, but the effect of opening the NALCN channels in time overrides the action on the K2P channels due to the large driving gradient of Na^+^ to enter the cell from a hyperpolarized state. A schematic on the effects of LPS related to the membrane potential in cells with normal expression of their endogenous K2P channels, likely of various subtypes, and overexpression of the ORK1-K2P subtype is highlighted in Figure 8.

Examining the various forms of LPS from different types of Gram-negative bacteria on various animal tissue models may well aid in understanding the direct cellular interactions of LPS on ion channels, receptors and secondary responses better [20], in addition to activating the CD14/TLR4/MD2 complex known in mammals. It is known that the LPS from *P. aeruginosa* and *S. marcescens* also hyperpolarize the skeletal muscle of crayfish [20,22] but has little effect on amphibian [22] or rodent skeletal muscle [22]; thus, the action of LPS may depend on the K2P subtype in these different preparations. In addition, LPS from *P. aeruginosa* and *S. marcescens* does not block glutamate receptors at the crayfish NMJ, but can even further enhance the action of 5-HT in promoting synaptic transmission at the NMJ [20,22]. It would be of interest to understand acute actions on neurons in mammals independent of secondary immune responses from cytokines, as this has still not been addressed in neurons in culture or in brain slices due to the presence of microglia in these preparations [22].

## 4. Materials and Methods

**Figure 9:**
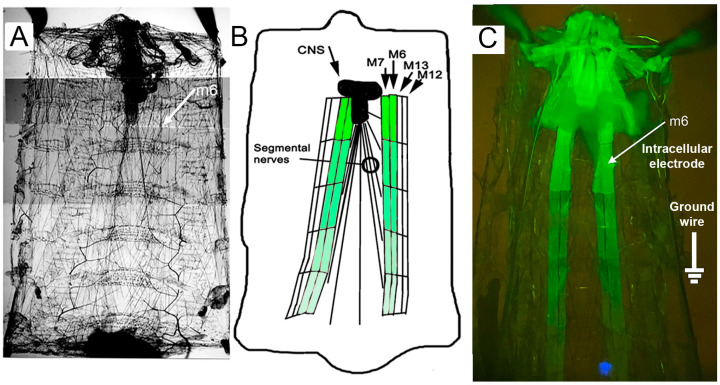
The larval preparation for recording membrane potential in defined m6 muscle fiber. (**A**) After dissection along the posterior longitudinal length of the larva and removing the internal organs except the nervous system and body wall muscles. (**B**) Schematic of the fileted preparation identifying the m6 and m7 muscle fibers shown in green for contrast. (**C**) GFP expression in m6 m7 and illustrating segment 2 for recording from m6.

Early third-instar *Drosophila* CS larvae were used (50–70 h) post-hatching. The larvae were maintained at room temperature, ~21 °C, in vials partially filled with a cornmeal–agar–dextrose–yeast medium. Overexpression of the ORK1receptor in larval body wall muscles (m6 and m7) was achieved by crossing homozygous males of BG487 (BDSC stock # 51634) with female virgins of UAS-ORK1 (BDSC stock # 6586). The female parental strain is referred to as UAS-ORK1 and the F1 generation is referred to as M6-M7 > ORK1. Males of BG487 were also crossed with female virgins of a non-conducting ORK1 transgene (BDSC stock#6587). Parental referred to as UAS-ORK1NC and the F1 generation is referred to as M6-M7 > ORK1NC. The non-conducting ORK1 serves as a control for the overexpression of protein. The two parental ORK1 strains were described earlier and both also co-express GFP [63] and the BG487 has a specific expression in muscles 6 and 7 [64,65]. The expression profile of the GFP and ORK1 and ORK1NC protein was illustrated by the GFP-loaded m6-m7 along the length of the larvae (Figure 9). These *Drosophila* strains were obtained from the Bloomington Drosophila Stock Center (BDSC).

The technique of dissecting larvae and measuring membrane potential was described previously [29]. All segmental nerves were transected close to the larval brain to prevent spontaneous evoked contractions induced from the CNS of the larvae. A novel dissection technique is described in Elliott and Cooper [29], which prevents damage to the m6 muscle fibers in segments 1 and 2 by providing a means of lifting the CNS and segmental nerves off the muscle fibers prior to cutting the nerves. The recording dish for the larvae are illustrated in video format [66]. In brief, the early third-instar larval *D. melanogaster* was dissected in physiological saline. Segment 1 has a unique muscle (i.e., m31) with a very high expression of the GFP marker, but this cell is easily damaged during larval dissection; however, the m6 muscle in segment 2 is reliably preserved during dissection for use in physiological studies and so it was used in these studies.

To monitor the transmembrane potentials of the body wall muscle (m6) of 3rd instar larvae, a sharp intracellular electrode (30 to 40 megaohm resistance) filled with 3 M KCl impaled the fiber. An Axoclamp 2B (Molecular Devices, Sunnyvale, CA, USA) amplifier and 1 X LU head stage were used. Data were collected using a PowerLab/4sp (ADInstruments, Colorado Springs, CO, USA), and analyzed with LabChart 7.0 (ADInstruments, Colorado Springs, CO, USA) which were recorded on a computer at a 20 kHz sampling rate along with the use of a NPI GMbH filter (type EPMS07 DPA 2F, from Adam and List Associate, LTD., 1100 Shames Drive, Westbury, NY 11590, USA) at low pass filtered at 3.0 kHz with no high pass filtering. The preparations were bathed in saline and then exchanged to one containing different concentration of KCl or LPS or a combination of the two, as described in the Results. A modified basal HL3 saline was used (NaCl 70 mM, KCl 5 mM, MgCl_2_·6H_2_O 20 mM, NaHCO_3_ 10 mM, Trehalose 5 mM, sucrose 115 mM, BES 25 mM, and CaCl_2_·2H_2_O 1 mM, pH 7.1; [50,51]). The pH of the saline was maintained at 7.2. The LPS compound used for assessment was *Serratia marcescens* (product number L6136; Sigma-Aldrich, St. Louis, MO, USA). The concentration of the LPS was 500 µg/mL in order to compare and contrast with prior studies [20,24,41,42,46,47]. In some protocols, N-methyl-D-glucamine (NMDG) was used to replace NaCl at the same concentration of 70 mM. The various forms of salines used are described in Table 1.

To assess the effects of LPS in the absence and presence of external K^+^ to drive the membrane to more negative values, Protocol 2 was used where the bathing saline was exchanged from Media 1 to Media 2 (Table 1, Figure 1). To examine the effect of reduced external Na^+^ and LPS, the NaCl was replaced with NMDG by exchanging Media 1 to Media 4 (Protocol 3, Table 1, Figure 1). Lastly, to examine the effect of reduced external K^+^ and external Na^+^ concentrations, the bathing media was first exchanged from Media 1 to Media 2 and then to Media 3 (Protocol 4, Table 1, Figure 1). The last bathing solution was then used with the presence of LPS for 5 min, followed by at least two bath exchanges to the same media without LPS.

The raw values of membrane potential are graphed for each preparation. Paired *t*-tests were used to compare changes in membrane potential for different conditions within a paradigm. Percentage changes in membrane potential were determined from the initial values in normal saline as compared to the different salines used to compare the effects within a group. This is a means to normalize preparations with varying membrane potentials. This was determined by the absolute difference in (initial–experimental) divided by the initial value and the resultant multiplied by 100 for the percentage change. The percentage change from the initial membrane potential to the lowest values when exposed to LPS was determined for each paradigm. The percentage change was also determined for the beginning values to a changed condition, or from when initially exposed to LPS to after 5 min of being exposed.

## Figures and Tables

**Table 1:** The composition of various salines used in different protocols. These are referred to as M1, M2, M3, and M4 in the text.

Compound	Media 1	Media 2	Media 3	Media 4
NaCl	70 mM	70 mM	0 mM	0 mM
NMDG	0 mM	0 mM	80 mM	70 mM
KCl	5 mM	0 mM	0 mM	5 mM
MgCl_2_·6H_2_O	20 mM	20 mM	20 mM	20 mM
NaHCO_3_	10 mM	10 mM	0 mM	10 mM
Trehalose	5 mM	5 mM	5 mM	5 mM
Sucrose	115 mM	115 mM	115 mM	115 mM
BES	25 mM	25 mM	25 mM	25 mM
CaCl_2_·2H_2_O	1 mM	1 mM	1 mM	1 mM
pH 7.2	NaOH	NaOH	HCl	HCl

## Data Availability

All data are presented in the graphs.
